# Exercise Participation Among Physically Active Adults: A Multidimensional Analysis of Demographic, Anthropometric, Personality, and Behavioral Factors

**DOI:** 10.3390/ijerph23020209

**Published:** 2026-02-08

**Authors:** Ioannis Tsartsapakis, Aglaia Zafeiroudi, Ioannis Trigonis, Charilaos Kouthouris

**Affiliations:** 1 Department of Physical Education & Sport Science at Serres, Aristotle University of Thessaloniki, 62100 Serres, Greece; 2 Department Physical Education & Sport Science, University of Thessaly, 42100 Trikala, Greece; azafeiroudi@uth.gr (A.Z.); kouthouris@uth.gr (C.K.); 3 Department of Physical Education and Sport Science, Democritus University of Thrace, 69100 Komotini, Greece; itrigon@phyed.duth.gr

**Keywords:** cultural variability, behavioral clustering, motivational orientation, exercise environments, public health equity, psychosocial profiling

## Abstract

**Highlights:**

**Public health relevance—How does this work relate to a public health issue?**

- Physical activity participation varies across demographic, anthropometric characteristics, and psychological characteristics, shaping how adults engage in active lifestyles.
- Understanding these differences supports more effective public health strategies to increase population-level activity.

**Public health significance—Why is this work of significance to public health?**

- BMI, weight history, and psychological traits influence exercise modality, duration, and motivational orientation.
- Identifying these determinants helps explain heterogeneity in physical activity behavior within adult populations.

**Public health implications—What are the key implications or messages for practitioners, policy makers and/or researchers in public health?**

- Tailored physical activity interventions should consider demographic, anthropometric, and psychological profiles to enhance adherence.
- Behavioral segmentation can guide targeted health promotion programs and support more efficient resource allocation.

**Abstract:**

Regular participation in recreational physical activity is a key determinant of population health, yet behavioral heterogeneity across adults remains insufficiently understood. This study examined how demographic, anthropometric, and psychological characteristics relate to exercise type (H1), weekly frequency (H2), daily duration (H3), motivational regulation (H4), personality-based differences in exercise modality (H5), and multidimensional behavioral clustering (H6) within a large sample of physically active recreationally active adults. A total of 1564 participants (age: M = 33.65, SD = 9.83 years) completed standardized questionnaires assessing physical activity behavior, body mass index, weight history, personality traits (Five-Factor Model), and motivational regulation (BREQ-2). Non-parametric tests, multinomial logistic regression, and cluster analysis were applied. Gender and age consistently predicted exercise frequency and duration (H1–H3), while higher BMI and reported weight problems were strongly associated with health- and appearance-related motives (H4). Personality traits were linked to exercise modality but showed limited associations with motivational regulation (H5), suggesting that activity preferences reflect relatively stable psychological profiles, whereas motives are more context-dependent. Cluster analysis identified three distinct behavioral profiles combining demographic, physical, and psychological attributes (H6), with meaningful differences in exercise modality, duration, and motivational orientation. These findings provide preliminary evidence for the utility of behavioral segmentation in recreational physical activity and highlight the potential of tailored public health strategies that account for demographic, anthropometric, and personality-based differences in engagement patterns.

## 1. Introduction

Regular participation in physical activity is widely recognized as a central determinant of population health and a cornerstone of global strategies for health promotion and disease prevention. International guidelines emphasize that adequate engagement in moderate-to-vigorous physical activity reduces the risk of non-communicable diseases, enhances psychological wellbeing, and improves overall quality of life [1]. Understanding the behavioral, demographic, and psychological factors that shape physical activity patterns is therefore essential for designing targeted public health interventions that support sustained engagement across diverse adult populations. Within this context, the present study examines how demographic, anthropometric, and psychological characteristics interact to influence recreational exercise behavior, with the aim of informing segmentation-based strategies relevant to public health planning.

Participation in physical activity is influenced by measurable demographic and physical factors, including age, gender, education, income, and body mass index (BMI). These variables determine both access to exercise opportunities and the intensity of engagement, and they provide a basis for empirical comparison across groups [2]. While broader sociological perspectives have emphasized cultural norms and social stratification [3,4], the present study acknowledges such perspectives only as theoretical background and does not adopt them as interpretive conclusions. Instead, the analytical focus remains strictly on quantifiable indicators, BMI, training behavior, education, income, and personality traits, that allow for empirical comparison across groups. This approach ensures alignment between the literature review and the study’s objectives, which are to analyze demographic, anthropometric, and psychological predictors of recreational activity.

In many countries, recreational exercise is shaped by lifestyle practices and evolving health behaviors, with participation spanning urban gyms, coastal promenades, rural trails, and community programs [5]. These modalities correspond to measurable differences in motivational orientation and body-related attitudes [6]. Outdoor settings have been associated with more positive body image and lower neuroticism, while indoor formats align with appearance-based motivation and eating attitudes. Regular engagement also correlates with adherence to healthier lifestyle patterns, including dietary behaviors [5]. Despite these insights, national-level data on exercise segmentation remain limited, and only a few studies have examined how demographic and anthropometric characteristics variables interact with behavioral clustering in specific populations [7]. This gap highlights the need for empirical segmentation studies that integrate demographic, anthropometric, and psychological predictors to clarify exercise participation patterns.

A multidimensional framework is required to understand participation in recreational physical activity, as demographic, anthropometric, and personality variables jointly shape behavioral outcomes. Demographic factors such as age, gender, education, employment status, and marital status influence both the frequency and type of recreational activity. Age operates as a biological determinant, with younger adults favoring high-intensity formats and older individuals prioritizing health maintenance and rehabilitation [8]. Gender differences are evident, as men often engage in competitive or strength-based activities, while women prefer group-based or wellness-oriented formats [9]. Education and employment shape access to facilities and time availability, influencing motivation and behavioral outcomes [10,11]. Marital status contributes to adherence, with married individuals reporting lower frequency but greater consistency in health-oriented routines [12]. These variables highlight the importance of demographic segmentation in understanding exercise participation [13].

Anthropometric indicators such as BMI, weight history, height, and waist-to-hip ratio also shape exercise behavior. Individuals with elevated BMI values often engage for weight management or health recovery [14], while those within normative ranges prioritize enjoyment, appearance, or performance. Greek data have shown that weight history and BMI are associated with distinct patterns of exercise and eating attitudes, influencing motivational orientation through internalized perceptions of health and body image [15]. Height and weight contribute to self-perception and social comparison processes within exercise settings, influencing both motivational orientation and modality selection [16]. These characteristics are closely linked to health-related behaviors and can inform targeted interventions for populations at elevated risk.

Exercise frequency and duration represent core behavioral outcomes shaped by individual motivation and external conditions. Weekly frequency is associated with demographic predictors, with higher adherence among individuals reporting greater self-efficacy and resilience [17]. Daily duration relates more closely to anthropometric factors, particularly BMI and perceived fitness. Elevated BMI values are linked to shorter sessions due to fatigue or reduced confidence, while normative or athletic profiles correspond to longer, performance-oriented routines [18]. These findings highlight the combined influence of psychological and physiological factors and support the design of targeted interventions [19]. A comprehensive understanding requires examining how frequency and duration vary with personality traits and demographic conditions [20].

Personality traits contribute to the prediction of exercise behavior, particularly in relation to activity type, frequency, and motivational orientation. The Five-Factor Model—extraversion, conscientiousness, neuroticism, openness, and agreeableness—has been widely applied in exercise psychology to explain individual variation in participation patterns [21]. Extraversion is linked to socially engaging or high-energy activities, conscientiousness to adherence and goal-directed behavior, and neuroticism to avoidance and lower engagement in structured programs [2]. Openness corresponds to preference for novel or outdoor formats, while agreeableness relates to cooperative and group-based modalities [6]. Studies have examined how these traits interact with body image and eating attitudes across exercise environments. Outdoor exercisers score higher in extraversion and conscientiousness and lower in neuroticism, with more positive body image and higher self-esteem [6]. A related review emphasized the role of personality in shaping health-related behaviors, showing that openness and conscientiousness are linked to healthier choices and greater consistency [22]. These findings suggest that personality traits influence both behavioral patterns and psychological outcomes. Segmenting populations based on personality may support the development of adaptive public health programs [23].

Exercise modality reflects personal preferences and contextual conditions. Activities such as running, cycling, swimming, and gym-based training differ in physical demands, social structure, and accessibility [24]. Group formats promote social bonding and shared goals, while solitary formats appeal to individuals who value autonomy or flexible scheduling. The physical setting also contributes to psychological outcomes. Outdoor environments are associated with improvements in mood, vitality, and stress reduction compared to indoor settings [25]. Participants report greater enjoyment, stronger engagement, and higher intent to repeat the activity when exercising outdoors, indicating that environmental context enhances behavioral sustainability [6].

The psychological motives that guide exercise participation include both internal and external influences. Individuals engage in recreational physical activity for reasons related to health, enjoyment, appearance, weight management, and rehabilitation [26]. According to self-determination theory, the degree to which these motives satisfy psychological needs for autonomy, competence, and relatedness shapes the sustainability of exercise behavior [27]. Health-related motives are more common among older adults and individuals with elevated BMI, who often prioritize disease prevention and functional mobility [28]. Appearance and performance motives are prevalent among younger individuals and those within normative weight ranges, often influenced by aesthetic ideals and competitive goals. Enjoyment and stress relief are reported across age groups, especially in recreational settings where intrinsic motivation dominates. Rehabilitation motives are typically linked to injury recovery or chronic condition management and are shaped by physiotherapeutic guidance [29]. Although motivational profiles vary, few studies have explored how these motives cluster or relate to personality and demographic variables [30].

Despite growing interest in personality and exercise behavior, segmentation frameworks that integrate psychological traits with demographic and anthropometric variables remain limited. Most studies examine these domains separately, reducing the ability to identify complex behavioral profiles. Recent segmentation of long-distance runners has demonstrated the utility of cluster analysis in capturing multidimensional behavioral typologies that combine demographic and psychological factors [7]. Typological analysis of older adults has revealed distinct motivational barriers and conditions for behavioral change, underscoring the relevance of segmentation in specific adult populations [31]. Findings from self-determination theory further support the integration of psychological variables in understanding exercise behavior, highlighting how autonomy, competence, and relatedness interact with demographic and physical characteristics to shape participation patterns [32]. These insights reinforce the need for multidimensional profiling, where exercise engagement has been studied in isolated domains but not within integrated frameworks.

Segmentation research that integrates psychological, demographic, and anthropometric variables remains scarce. Existing studies have explored exercise behavior in relation to lifestyle and motivational factors, yet empirical applications of cluster analysis are limited. A recent study by Terry et al. [33] applied mood-based clustering, demonstrating the feasibility of psychosocial segmentation. However, most segmentation efforts continue to rely on descriptive typologies or single-variable approaches, without capturing the multidimensional interplay of demographic, physical, and psychological factors. The diversity of exercise modalities, from recreational walking and cycling to structured gym-based programs, presents an opportunity for segmentation approaches that inform personalized programming and targeted public health strategies [34].

The present study responds to this gap by examining how demographic characteristics, anthropometric indicators, and personality traits relate to exercise type, weekly frequency, daily duration, and motivational orientation. Through cluster analysis, distinct behavioral profiles are identified based on combinations of demographic, physical, and psychological variables. This segmentation informs interventions that promote sustained participation across diverse groups. By integrating psychological traits with demographic and anthropometric data, the study advances understanding of exercise engagement as a multidimensional phenomenon.

Research Hypotheses:

**H1.**  *Age, gender, education, employment, and marital status are expected to differentiate exercise frequency and duration.*

**H2.**  *Body mass index, height, weight, and weight history are anticipated to influence the main motive for physical activity, especially regarding health, appearance, weight control, and rehabilitation.*

**H3.**  *Personality traits, measured via the Five-Factor Model, are hypothesized to relate to exercise type and motivational orientation.*

**H4.**  *Combined demographic, anthropometric, and psychological variables are expected to predict the main reason for participation, enabling deeper insight into motivational patterns.*

**H5.**  *The same multidimensional set is expected to predict exercise modality preference, including cycling, running, swimming, indoor training, or non-participation.*

**H6.**  *Cluster analysis is expected to reveal distinct behavioral profiles based on integrated demographic, anthropometric, psychological, and behavioral patterns.*

## 2. Materials and Methods

### 2.1. Participants

The study sample consisted of 1564 adults aged 18–59 years (mean [M] = 33.65, standard deviation [SD] = 9.83), recruited through convenience sampling from multiple regions in Greece. The age range was selected to capture variation across young, middle-aged, and older participants within the adult age range, acknowledging that younger individuals tend to be more active in daily life, with young adults defined as 18–34 years, middle-aged adults as 35–54 years, and older adults as 55–59 years. Eligibility criteria required regular engagement in recreational physical activity, defined according to international guidelines [34,35] as at least 150 min of moderate-intensity or 75 min of vigorous-intensity activity per week. This criterion ensured that all participants met minimum recommended levels of physical activity exposure. All participants were current exercisers; therefore, the study focuses on segmentation within active populations and does not address inactivity or exclusion. Twenty-one questionnaires with missing data were excluded from analysis. The research protocol was approved by the Internal Ethics Committee of the Department of Physical Education and Sport Science, University of Thessaly, Greece (2252, 3-2/11 October 2023). 

Descriptive distributions for gender, education level, marital and employment status, reported weight problems, exercise type, frequency, and duration are presented in Table 1.

### 2.2. Procedures

Participants completed three standardized instruments assessing behavioral and psychological dimensions relevant to the study. The first was a structured questionnaire developed specifically for the research, which included items on gender, age, height, and weight. Body mass index (BMI) was calculated using self-reported anthropometric data. In addition to these physical measures, participants provided information on their preferred type of exercise, using an expanded taxonomy that included fitness (structured aerobic and high-intensity interval training [HIIT] programs typically performed in gym settings, such as Tabata, circuit training, and cross-training), running, cycling, swimming, walking, hiking, yoga, dance, team sports, martial arts, and an open-ended “other” category.

Motivation was assessed using the Behavioral Regulation in Exercise Questionnaire-2 (BREQ-2), the second version of the BREQ series, which captures intrinsic regulation, identified regulation, introjected regulation, external regulation, and amotivation [35,36]. The Greek adaptation of the BREQ-2 has demonstrated satisfactory psychological properties, including high internal consistency and construct validity, confirming its suitability for use in Greek populations [37]. Each subscale score was computed as the mean of its corresponding items, with responses rated on a five-point scale ranging from 0 (“not true for me”) to 4 (“very true for me”). The five subscale scores were treated as continuous variables and entered directly into the statistical analyses as indicators of distinct forms of motivational regulation. This instrument provides a multidimensional assessment of motivational orientation, allowing participants to indicate multiple motives and their relative strength.

The third instrument was the Traits Personality Questionnaire 5 (TPQue-5), a validated Greek language measure of the Five Factor Model of personality. The TPQue-5 consists of 101 items, including 75 statements assessing neuroticism, extraversion, agreeableness, conscientiousness, and openness to experience, and an additional 26 items measuring social desirability. Responses were recorded on a five-point Likert scale ranging from complete disagreement to complete agreement. The instrument is suitable for individuals aged 17 and older and has demonstrated high internal consistency and satisfactory test–retest reliability in Greek samples. Its psychological properties were established by Tsaousis and colleagues [38,39], and it represents an abbreviated adaptation of the Revised NEO Personality Inventory [40]. Scoring followed the standard procedure described by Tsaousis, whereby item responses for each personality dimension were reverse-coded where appropriate, summed, and averaged to produce five continuous subscale scores. These subscale scores were used as the grouped outcome variables in the analyses.

All TPQue-5 and BREQ-2 subscale scores were treated as continuous composite variables, consistent with standard psychological practice.

### 2.3. Data Analysis

All statistical analyses were performed using IBM SPSS Statistics, version 29.0 (IBM Corp., Armonk, NY, USA). Prior to analysis, the dataset was reviewed for completeness and internal consistency. Twenty-one participants with substantial missing data (more than 20% of items unanswered across the instruments) were removed from the analytic dataset. For the remaining participants, missing values were handled using listwise deletion at the analysis level. Descriptive statistics were computed for all variables. For continuous variables (age, height, weight, BMI, exercise frequency and duration, and the five psychological dimensions assessed by the TPQue5), means, standard deviations, and minimum and maximum values were calculated. For categorical variables (gender, marital status, educational level, employment status, weight history, exercise type, and primary exercise motives), frequency distributions and percentages were computed.

The Kolmogorov–Smirnov test indicated deviations from normality for several continuous variables; therefore, non-parametric statistical methods were used for all subsequent analyses. Hypothesis H1 examined whether demographic variables influenced exercise frequency and duration using Kruskal–Wallis and Mann–Whitney U tests. Hypothesis H2 investigated associations between anthropometric indicators and participants’ primary exercise motives. Hypothesis H3 explored associations between psychological traits and both exercise type and motive using Kruskal–Wallis tests.

Spearman’s rank correlation coefficients were calculated to assess associations among continuous variables. Hypotheses H4 and H5 were tested using multinomial logistic regression to determine whether demographic, anthropometric, and psychological characteristics predicted exercise motives and exercise type.

Finally, Hypothesis H6 was examined using the Two-Step Cluster Analysis procedure implemented in IBM SPSS Statistics, which allows the simultaneous inclusion of continuous and categorical variables. Continuous variables were normalized using min–max scaling, and categorical variables were dummy-coded. The clustering procedure was conducted on the full sample of 1543 participants after removing a follow-up weight-related variable that was not applicable to all respondents. The number of clusters was determined using the Bayesian Information Criterion (BIC) and the silhouette measure of cohesion and separation. Post hoc comparisons across clusters were performed using Kruskal–Wallis tests for continuous variables and chi-square tests for categorical variables, with effect sizes reported where appropriate.

## 3. Results

### 3.1. Descriptive Analysis

To establish a foundational understanding of the sample, descriptive statistics were computed for continuous variables (age, height, weight, BMI, and the five TPQue-5 personality dimensions), as well as for ordinal indices of weekly frequency and daily duration. Descriptive statistics were also calculated for the five BREQ-2 motivational subscales (intrinsic regulation, identified regulation, introjected regulation, external regulation, and amotivation) to provide a comprehensive overview of motivational orientation within the sample. Means and standard deviations are summarized in Table 2 before inferential analysis. The recruited sample covered the full intended age range (18–59 years), with representation across young, middle-aged, and older adults. The distribution was not uniform, as younger adults constituted the largest proportion of the sample, reflecting typical participation patterns in recreational exercise settings.

### 3.2. Assessment of Distributional Assumptions

Normality tests were not applied to categorical or ordinal variables due to their measurement level. For continuous variables, the use of non-parametric methods in subsequent analyses was justified by the presence of ranked outcomes (ordinal frequency and duration) and the aim to compare groups across mixed measurement levels. Robust parametric alternatives were considered less appropriate given the predominance of ordinal dependent variables in H1–H3.

### 3.3. Reliability Analysis

Internal consistency for the TPQue-5 dimensions was acceptable (Cronbach’s α = 0.71–0.84), indicating stable measurement of the five personality factors within the sample. Reliability analysis for the Behavioral Regulation in Exercise Questionnaire-2 (BREQ-2) showed satisfactory internal consistency across its five subscales (intrinsic regulation, identified regulation, introjected regulation, external regulation, and amotivation), with Cronbach’s α values ranging from 0.72 to 0.86. These results confirm that both instruments demonstrated stable and coherent measurement properties in the present sample, supporting their suitability for assessing personality traits and motivational regulation in exercise contexts.

### 3.4. Influence of Demographic Variables on Exercise Frequency and Duration (H1)

Group comparisons were conducted using Mann–Whitney U tests for gender and Kruskal–Wallis tests for education and employment status. Spearman’s rank correlations were used to examine associations between age and both exercise frequency and duration. Statistically significant differences were primarily observed for daily duration, with gender emerging as the most consistent predictor. Educational level and employment status showed significant effects on duration but not on frequency. Age demonstrated a weak positive correlation with duration.

Results are consolidated in Table 3, with interpretive text presented below the table in accordance with reviewer recommendations.

Gender differences were statistically significant for both weekly frequency (*U* = 248,291.500, *p* < 0.001, r = 0.153) and daily duration (*U* = 236,544.500, *p* < 0.001, *r* = 0.202). Men reported higher ranks for both outcomes, indicating greater weekly frequency and longer daily sessions. Educational level was associated with statistically significant differences in weekly frequency (*H* = 11.762, df = 5, *p* = 0.038, η^2^ = 0.0076) and daily duration (*H* = 16.070, *df* = 5, *p* = 0.007, η^2^ = 0.0104), although effect sizes were small. Employment status also showed significant differences in weekly frequency (*H* = 29.157, df = 8, *p* < 0.001, η^2^ = 0.0189) and daily duration (*H* = 44.566, *df* = 8, *p* < 0.001, η^2^ = 0.0289), with small-to-moderate effect sizes. Age was not significantly correlated with weekly frequency (ρ = 0.038, *p* = 0.138), but a weak positive correlation was observed with daily duration (ρ = 0.057, *p* = 0.026), suggesting that older participants reported slightly longer exercise sessions.

### 3.5. Influence of Anthropometric Characteristics Indicators on Exercise Motivation (H2)

To examine the influence of anthropometric characteristics indicators on motivational orientation, body mass index and weight history were analyzed in relation to exercise motives. Results are summarized in Table 4. Body mass index was significantly associated with exercise motives (*H* = 160.30, *df* = 4, *p* < 0.001, η^2^ = 0.10). Participants reporting weight loss as their primary motive had the highest ranks, followed by rehabilitation and health, whereas appearance and enjoyment were associated with lower BMI values. Weight history also differentiated motives (*U* = 185,774.00, *Z* = −8.28, *p* < 0.001, *r* = 0.21), with individuals reporting weight-related problems more likely to endorse appearance and weight-loss motives. Effect sizes were moderate, indicating meaningful anthropometric characteristics influences on motivational orientation.

These findings should be interpreted with caution, as motivational orientation was assessed through a single forced-choice item rather than a validated multi-item questionnaire, representing a methodological limitation.

### 3.6. Personality Traits Across Exercise Types and Motives (H3)

To examine whether personality traits relate to exercise type, Kruskal–Wallis tests were conducted across the five TPQue-5 dimensions. Results are summarized in Table 5 and Table 6. Statistically significant differences were observed across exercise types, whereas no significant differences emerged across motivational categories. The *H* values indicated that neuroticism showed the strongest differentiation, with swimmers exhibiting the highest ranks and cyclists the lowest. Extraversion, agreeableness, and conscientiousness were highest among cyclists, while runners displayed slightly higher openness. Effect sizes were calculated for statistically significant comparisons across exercise types. Neuroticism showed the strongest differentiation (η^2^ = 0.032), followed by extraversion (η^2^ = 0.014), agreeableness (η^2^ = 0.015), and conscientiousness (η^2^ = 0.015), all indicating small practical effects.

To examine whether personality traits relate to motivational orientation, Kruskal–Wallis tests were conducted across the five TPQue-5 dimensions. Results are summarized in Table 7. No statistically significant differences were observed across motive categories, indicating that personality traits were not associated with primary exercise motives. All H values were non-significant (*p* > 0.05), suggesting that motivational orientation was independent of trait variation. No effect sizes were computed for exercise motivation, as none of the personality traits reached statistical significance.

### 3.7. Predictors of Exercise Motivation (H4)

To evaluate whether demographic and anthropometric variables predict motivational orientation, a multinomial logistic regression was performed using enjoyment as the reference category. The overall model was statistically significant (*χ^2^* = 643.93, df = 180, *p* < 0.001), with a Nagelkerke *R*^2^ of 0.391, indicating moderate explanatory strength. Weight history emerged as the strongest predictor, with individuals reporting weight-related problems being substantially more likely to endorse weight-loss and appearance motives. Age and gender also contributed, with younger participants and women showing higher odds of weight-loss and appearance motives compared to enjoyment.

Daily exercise duration consistently differentiated motives, with shorter sessions associated with weight-loss, appearance, and health motives. Weekly frequency predicted health motives, with fewer sessions linked to health orientation. Other anthropometric indicators (height, weight, BMI) and personality traits did not reach statistical significance. These findings suggest that motivational orientation is primarily shaped by anthropometric characteristics and demographic characteristics, particularly weight history, age, gender, and exercise duration.

### 3.8. Predictors of Exercise Type (H5)

To examine whether demographic, anthropometric, and personality variables predict exercise modality, a multinomial logistic regression was conducted with swimming set as the reference category. The non-participation category was removed, as the sample did not include non-exercisers. The overall model demonstrated satisfactory fit (Nagelkerke *R*^2^ = 0.301), indicating moderate explanatory strength.

Age reduced the likelihood of selecting cycling (*OR* = 0.779, *p* = 0.030) and fitness (*OR* = 0.555, *p* < 0.001) compared to swimming. Body mass index increased the odds of choosing fitness (*OR* = 1.391, *p* = 0.003) and reduced the odds of running (*OR* = 0.714, *p* = 0.003). Neuroticism consistently lowered the likelihood of cycling, running, and fitness participation (all *p* < 0.001), whereas agreeableness increased the odds of cycling (*OR* = 1.324, *p* = 0.004). Gender effects were evident, with men more likely to choose running (*OR* = 1.566, *p* = 0.046) and less likely to choose fitness (*OR* = 0.161, *p* < 0.001). Employment status also contributed, with fitness favored by students and employed individuals (*p* = 0.018).

Other predictors, including height, weight, and the remaining personality traits, did not reach statistical significance. These findings suggest that exercise modality preferences are shaped by a combination of demographic and psychological factors, particularly age, BMI, neuroticism, agreeableness, gender, and employment status.

Effect sizes were evaluated through Nagelkerke *R*^2^ and odds ratios. The model demonstrated moderate explanatory power (*R*^2^ = 0.301), accounting for approximately 30% of the variance in exercise type. Gender showed the strongest predictive effect, with men significantly less likely to choose fitness (Exp(B) = 0.161) and more likely to choose running (Exp(B) = 1.566). Neuroticism and agreeableness also influenced modality selection, with moderate effects observed for cycling. BMI and age contributed modestly to modality differentiation, while education level showed no significant impact.

### 3.9. Behavioral Profiling: Clustering Method Reconsideration (H6)

To identify behavioral profiles of exercisers, a TwoStep Cluster analysis was conducted using mixed data (demographic, anthropometric, psychological, and behavioral variables). The procedure yielded three clusters. Model quality was limited (silhouette measure ≈ 0.20; BIC-based selection), indicating weak separation. Nevertheless, the three-cluster solution was retained due to its interpretability and the large sample size. Cluster sizes were 695 (45.0%), 535 (34.7%), and 313 (20.3%) participants, with detailed composition presented in Table 8.

Cluster characteristics indicated clear demographic differentiation. Cluster 1 comprised predominantly older men, with higher body weight, higher BMI, and longer daily exercise duration, and showed greater representation in cycling and running. Cluster 2 included almost exclusively women, representing a mid-aged group with lower BMI and a strong preference for fitness activities. Cluster 3 consisted largely of younger individuals, mostly students, with lower BMI and mixed engagement in fitness and running. Personality traits showed minimal differentiation across clusters, consistent with prior null findings.

Motivational orientation differed significantly across clusters (χ^2^ values and effect sizes recalculated for the updated sample; see Table 9). Cluster 1 showed a relatively higher representation of health-related motives compared to the other clusters. Cluster 2 favored appearance-related and enjoyment motives, and Cluster 3 presented a more balanced distribution including rehabilitation motives.

Exercise type preferences also varied significantly across clusters (χ^2^ values and effect sizes recalculated; see Table 10). Cluster 1 was dominated by cycling and running, Cluster 2 by fitness, and Cluster 3 showed moderate representation across fitness and running.

These results suggest that although cluster separation was statistically weak, meaningful behavioral and demographic differentiation emerged across clusters. Interpretations are presented cautiously, avoiding psychological labeling, and effect sizes are reported to indicate the magnitude of differences.

Effect sizes were calculated using Cramér’s V for categorical comparisons across clusters. Motive distribution yielded a small-to-moderate effect (*V* = 0.203), while exercise type preferences showed a moderate effect (*V* = 0.341), indicating meaningful differentiation of behavioral patterns across psychosocial profiles.

## 4. Discussion

Exercise participation remains a central public health priority, particularly in contexts where physical activity levels vary substantially across demographic groups and gender disparities persist. Within this framework, the present study examined how demographic, anthropometric, and psychological characteristics relate to exercise modality, frequency, duration, and motivational orientation. By integrating these variables into a multidimensional framework and applying cluster analysis, the study identified coherent behavioral profiles that enhance understanding of physical activity engagement. The findings confirm established associations, reveal new patterns, and demonstrate the value of segmentation approaches for public health research and targeted intervention design.

The first hypothesis (H1) was supported. Demographic variables, especially gender, education, employment status, and age, significantly influenced exercise behavior, with effects more pronounced for duration than frequency. Gender emerged as the most consistent predictor, with men reporting higher weekly frequency and longer daily sessions. This pattern aligns with findings from the Global Flourishing Study, which documented higher physical activity levels among men across 22 countries [41]. Although statistically significant, gender differences were modest, suggesting that while gender shapes opportunity and preference, its practical impact may be limited in populations already engaged in regular exercise.

Educational level did not affect exercise frequency but significantly influenced duration, with individuals holding secondary education reporting the longest sessions. This finding contrasts with studies from Malaysia, where higher education was associated with increased participation in structured physical activity [42]. Employment status also shaped duration, with unemployed or non-working participants reporting the longest sessions. The absence of time constraints may have enabled longer engagement, though the motivational basis for this pattern requires further investigation. Age was not significantly correlated with exercise frequency but showed a weak positive association with duration, suggesting that older adults may engage in longer sessions, consistent with cross-national evidence that older individuals prioritize sustained activity for health maintenance [41]. Taken together, these findings indicate that demographic variables contribute meaningfully to behavioral differentiation, though their explanatory power is moderate and context-dependent.

The second hypothesis (H2) was supported. Anthropometric characteristics indicators, particularly BMI and weight history, significantly influenced motivational orientation. Individuals with elevated BMI values were more likely to report external motives such as weight loss and appearance enhancement, whereas those with lower or normative BMI scores cited health promotion and enjoyment. These findings are consistent with previous research in Western populations, where BMI has been shown to correlate with extrinsically oriented exercise motives. Ingledew and Sullivan [43] demonstrated that individuals with higher body mass tend to prioritize appearance and weight control, while those with lower BMI are more likely to report intrinsic motives such as enjoyment and psychological well-being. Similarly, Swami et al. [44] found that internalization of the thin ideal was associated with increased emphasis on body-focused exercise goals, particularly among women.

Weight history also emerged as a significant predictor of motivational orientation. Participants with prior weight-related challenges were more likely to report appearance and weight loss as their primary motives, whereas those without such history emphasized health and psychological benefits. This distinction aligns with findings by Puhl and Heuer [45] who showed that individuals with a history of obesity often engage in exercise as a form of self-management. The present data extend this association by demonstrating that BMI retains predictive value even when controlling for other variables, suggesting that anthropometric characteristics status functions as a salient cue in shaping motivational orientation. From a theoretical standpoint, this pattern resonates with expectancy-value models of motivation, which posit that individuals engage in behaviors they perceive as both valuable and attainable [46].

However, not all studies converge on this interpretation. Sabiston et al. [47] observed that among adolescents with elevated BMI, motives for exercise were not exclusively appearance-driven but also included psychological empowerment and social integration. This suggests that body-related motives may be modulated by age, gender, and developmental context. Accordingly, the present findings should be interpreted within the specific age composition of the sample. Overall, BMI and weight history emerged as strong predictors of motivational orientation, underscoring the importance of anthropometric characteristics indicators in shaping exercise motives.

The third hypothesis (H3) was partially supported. Personality traits were significantly associated with exercise type but not with motivational orientation. Individuals scoring higher in extraversion, agreeableness, and conscientiousness were more likely to engage in cycling and running, while elevated neuroticism was observed among swimmers and fitness participants. These findings suggest that modality preference may reflect psychological dispositions. This pattern aligns with previous research indicating that personality traits influence behavioral preferences in physical activity. Shuai et al. [48] reported that extraversion and conscientiousness were positively associated with participation in competitive and socially interactive sports, while neuroticism was linked to solitary or low-intensity modalities. Similarly, Yang et al. [49] found that extraversion and conscientiousness predicted higher exercise frequency and adherence, although their impact on modality selection was less pronounced.

In the present study, the differentiation across exercise types was statistically significant but modest in magnitude. Effect sizes ranged from η^2^ = 0.006 to 0.030, with neuroticism showing the strongest differentiation across modalities. These values suggest that although psychological dispositions influence modality preference, their practical impact remains limited. The lack of association between personality traits and motivational orientation indicates that motives for exercise may be shaped less by stable traits and more by situational or behavioral factors. Hoyt et al. [50] argued that personality influences exercise behavior indirectly through attitudes and perceived behavioral control, rather than directly determining motivational categories. This interpretation is supported by the present findings, where mean rank differences across motive groups failed to reach statistical significance. Taken together, these results indicate that personality traits are relevant for exercise type but not for motivational orientation.

The fourth hypothesis (H4) was partially supported. Combined demographic and anthropometric characteristics, and personality variables contributed to the prediction of exercise motivation, though with uneven explanatory strength across models. In the first regression model, BMI emerged as the only consistent predictor, while personality traits failed to demonstrate significant influence. In the second model, which excluded undefined responses and used appearance as the reference category, explanatory power increased substantially, and additional predictors such as age, daily exercise duration, motivational orientation, and cluster-defined profiles reached statistical significance. The explanatory power of the second regression model was substantial, with Nagelkerke *R*^2^ = 0.752, indicating that approximately 75% of the variance in exercise motivation was accounted for by the included predictors.

The limited predictive value of personality traits aligns with previous research indicating that dispositional characteristics may influence exercise behavior indirectly, rather than determining specific motives. Hoyt et al. [50] demonstrated that personality traits operate through attitudinal and normative pathways, shaping intention rather than motive. Similarly, Yang et al. [49] found that personality traits predicted exercise adherence and self-efficacy, but not the underlying reasons for participation. Emm-Collison et al. [51] further showed that motivational profiles are dynamic and context-sensitive, with personality playing a limited role in transitions between motive categories. These findings reinforce the view that motivational orientation is shaped more by behavioral and anthropometric characteristics than by psychological disposition.

The fifth hypothesis (H5) was supported. Demographic and anthropometric characteristics, and psychological characteristics significantly predicted exercise modality preference. Age and BMI emerged as central predictors, with younger individuals favoring cycling and fitness, and those with higher BMI values gravitating toward indoor formats. These findings are consistent with Ball et al. [52], who emphasized the role of body composition in shaping comfort and accessibility within structured exercise settings. Emm-Collison et al. [51] also noted that age and BMI differentiate motivational profiles and behavioral patterns, especially in transitional life stages.

Neuroticism was negatively associated with all exercise types, reinforcing its role as a psychological barrier to engagement. Allen et al. [53] and Ronca et al. [54] found that individuals with elevated neuroticism experience heightened anxiety and self-consciousness in public or performance-based contexts, leading to avoidance or preference for solitary formats. In the present study, this trait consistently reduced the likelihood of choosing cycling, running, or fitness, suggesting that emotional instability may inhibit participation in socially exposed or physically demanding modalities. Agreeableness was positively linked to cycling, indicating that cooperative formats appeal to individuals with prosocial tendencies. This aligns with findings from Rhodes and Smith [2], who showed that interpersonal orientation influences preference for group-based or outdoor activities. A recent study from University College London [55] further demonstrated that personality traits predict not only exercise adherence but also enjoyment and stress reduction, with extroverts favoring high-intensity formats and neurotic individuals preferring short, private sessions.

Gender differences were also evident, with men more likely to choose running and women favoring structured aerobic and high-intensity interval training (HIIT) programs typically performed in gym settings. Lauderdale et al. [55] documented similar patterns. Employment status showed modest effects, with students and employed individuals more likely to engage in these gym-based aerobic and HIIT activities. Ahsan et al. [11] noted that occupational status influences both opportunity and motivation for structured exercise, particularly in urban populations. Educational level, however, did not significantly predict exercise type, suggesting that modality preference may be shaped more by psychological and physical factors than by formal education. The overall explanatory power of the regression model was moderate (Nagelkerke *R*^2^ = 0.301), indicating that approximately 30% of the variance in exercise type was accounted for by the included predictors. Effect sizes derived from odds ratios revealed that gender and neuroticism exerted the strongest influence, with men significantly less likely to choose these structured aerobic and HIIT programs and individuals with elevated neuroticism consistently avoiding socially exposed modalities. Other predictors such as age and BMI showed modest but interpretable effects.

The sixth hypothesis (H6) was confirmed. Cluster analysis revealed three distinct behavioral profiles integrating demographic, anthropometric, psychological, and behavioral dimensions. Cluster 1 consisted predominantly of older men with higher body weight and BMI, longer daily exercise duration, and a strong preference for outdoor endurance-based activities such as cycling and running. Their motivational profile showed elevated health-related and appearance-related motives. Comparable findings have been reported in adult fitness populations, where higher neuroticism and weight-related concerns are associated with increased exercise engagement but reduced enjoyment [54,56].

Cluster 2 comprised almost exclusively women, representing a mid-aged group with lower BMI and shorter exercise duration. Their personality scores were generally similar to the other clusters, and their motivational profile reflected a combination of appearance-related and enjoyment-based motives. This pattern aligns with self-determination theory and person-centered models of motivational regulation, which emphasize the coexistence of intrinsic and extrinsic drivers in recreational exercise contexts [26,57].

Cluster 3 included younger participants, largely students, with the lowest BMI and moderate exercise duration. They showed a mixed preference for fitness and running and reported a balanced motivational profile that included health-related motives and rehabilitation-oriented reasons. Similar profiles have been observed in younger and emerging-adult populations, where conscientiousness and openness contribute to sustained participation in structured health-oriented programs [58,59].

Clusters were differentiated by both motive and modality. Cluster 1 was more engaged in outdoor activities such as cycling and running, Cluster 2 showed a strong preference for indoor fitness formats, and Cluster 3 demonstrated mixed participation across fitness and running. Effect size analysis supported these differences, with Cramér’s *V* = 0.203 for motive distribution and V = 0.341 for exercise type preferences, indicating meaningful segmentation across profiles.

The confirmation of H6 highlights the value of integrated behavioral profiling. Rather than focusing on isolated predictors, the analysis identified coherent patterns that combine psychological traits, physical attributes, and exercise behavior. These clusters highlight measurable differences in motives and modalities that can inform tailored interventions, although the weak statistical separation warrants cautious interpretation. Cluster-specific implications can be drawn: Cluster 1 may benefit from programs that integrate emotional regulation strategies and body-image resilience; Cluster 2 may respond best to flexible formats that reinforce autonomy and enjoyment; and Cluster 3 may require structured interventions emphasizing routine reinforcement and health education [19,41].

### 4.1. Future Directions

Future research should investigate the longitudinal stability of the identified clusters and their responsiveness to tailored interventions, clarifying whether they represent enduring dispositions or transitional states. Extending the analysis to include additional variables directly measurable in survey or observational designs could improve the generalizability of the findings. Methodologically, approaches such as latent profile analysis, mixed-method designs, or longitudinal modeling may provide deeper insight into the dynamics of behavioral segmentation and capture changes in motivation and engagement over time. These directions highlight the potential of multidimensional profiling to advance exercise science by refining theoretical models and informing strategies that reflect the diversity of participant experiences.

### 4.2. Methodological Considerations and Limitations

Several methodological limitations should be acknowledged when interpreting the findings. First, the use of convenience sampling restricts the generalizability of the results beyond the specific adult population recruited in Greece. Although the sample size was adequate for multivariate analysis, its regional and self-selected nature may introduce bias, particularly in terms of exercise engagement and digital literacy. Second, the online administration of the survey may have excluded individuals without stable internet access or digital familiarity, potentially skewing the sample toward younger, urban, or more educated participants. Third, all data were self-reported, including anthropometric indicators and exercise behavior, which may be affected by social desirability or recall bias. While the TPQue-5 includes a social desirability scale, no statistical correction was applied to adjust for response distortion. This may limit the accuracy of personality and motivation-related findings.

Fourth, although motivational orientation was assessed using the validated multi-item BREQ-2 instrument, the study also included a single categorical item capturing participants’ primary motive. This item did not allow multiple or hierarchical responses, which may have constrained the interpretive sensitivity of analyses involving primary motive selection. Future studies should consider multi-response formats or scaled items to capture the multidimensional nature of exercise motivation more fully.

Fifth, the regression models did not initially include formal diagnostics for multicollinearity among predictors. Although the variables were theoretically distinct, the absence of variance inflation factor (VIF) or tolerance checks may raise concerns about overlapping variance and inflated coefficients. Supplementary diagnostics conducted post hoc indicated acceptable levels of multicollinearity (VIF < 3.0; Tolerance > 0.33), supporting the statistical integrity of the regression models.

Sixth, the clustering procedure relied on k-means analysis with Euclidean distance, which assumes spherical clusters and equal variance across dimensions. While the elbow method supported the three-cluster solution, no comparative analysis was initially conducted using alternative algorithms such as latent profile analysis or hierarchical clustering. A supplementary hierarchical cluster analysis using Ward’s method and squared Euclidean distance confirmed a three-cluster solution, enhancing the structural validity of the segmentation.

Seventh, the cross-sectional design precludes causal inference. Associations between personality traits, exercise motives, and modality preferences reflect correlational patterns and cannot be interpreted as directional or predictive over time. Finally, the exclusion of 45 incomplete cases, although necessary for statistical integrity, may have reduced variability in key variables, particularly among less engaged or more vulnerable participants. Given that all participants met the physical activity guidelines, the findings should be interpreted within the context of an active population, which limits generalizability to less active or sedentary groups.

## 5. Conclusions

Cluster analysis identified three distinct behavioral profiles that combined demographic, physical, and psychological attributes. These clusters differed in both exercise motives and modality preferences, offering an exploratory segmentation of engagement patterns within the sample. Although the differentiation across clusters was meaningful, the cross-sectional design, reliance on self-reported data, and single-item measurement of motives limit the generalizability of the findings.

The conclusions should therefore be interpreted as hypothesis generating rather than definitive. The study highlights potential associations between demographic, anthropometric characteristics, and psychological factors and exercise behavior, but further research is needed to confirm the stability of these profiles and to assess their applicability in broader or more representative populations. Longitudinal designs, validated multi-item measures of motivation, and alternative clustering approaches could strengthen future investigations and clarify the mechanisms underlying behavioral segmentation.

Overall, the findings provide preliminary evidence that integrated profiling may offer useful insights into exercise participation, particularly in contexts where low activity levels and gender disparities remain public health concerns. While interpretations are constrained by methodological limitations, the results represent an initial step toward more comprehensive models of behavioral segmentation based on measurable demographic, anthropometric, personality, and behavioral data.

## Figures and Tables

**Table 1:** Demographic, social, and behavioral characteristics of participants (%).

Variable	Groups	%	Variable	Groups	%
Gender	Male	52.1	Marital Status	Single/Divorced	62.4
	Female	47.9		Married	37.4
Education Level	Secondary	28.5	Employment	Student	19.2
	Tertiary	57.4		Employed	46.0
	Postgraduate	14.1		Unemployed/Other	34.8
Weight Problem	Yes	27.8	Exercise Type	Fitness	35.6
	No	72.2		Running	31.1
				Cycling	21.2
				Swimming	12.1
Weekly Exercise Frequency	1–2 sessions	9.7	Daily Exercise Duration	1 h	21.1
	3–4 sessions	41.1		Up to 2 h	60.7
	4–5 sessions	38.2		Up to 3 h	16.1
	>5 sessions	11.0		≥4 h	2.1

Note: Percentages are based on *N* = 1543 valid cases (no missing data).

**Table 2:** Descriptive Statistics for Continuous Variables and Composite Indices (*N* = 1543).

Variable	Minimum	Maximum	Mean	Standard Deviation
Age (years)	18	59	33.65	9.83
Height (meters)	1.54	1.99	1.74	0.08
Weight (kilograms)	43.0	140.00	70.76	13.21
Body Mass Index (BMI)	16.5	41.35	23.13	3.24
Extraversion (TPQue5)	25.0	74.00	51.88	7.80
Neuroticism (TPQue5)	15.0	72.00	41.33	9.78
Openness (TPQue5)	24.0	70.00	49.12	7.45
Agreeableness (TPQue5)	24.0	72.00	52.55	6.77
Conscientiousness (TPQue5)	24.0	73.00	50.72	7.38
Intrinsic Regulation (BREQ-2)	1.00	5.00	3.85	0.72
Identified Regulation (BREQ-2)	1.00	5.00	3.67	0.81
Introjected Regulation (BREQ-2)	1.00	5.00	2.91	0.76
External Regulation (BREQ-2)	1.00	5.00	2.45	0.69
Amotivation (BREQ-2)	1.00	5.00	1.88	0.65

**Table 3:** Non-parametric tests for demographic influences on weekly exercise frequency and daily exercise duration (*N* = 1543).

Variable	Test Type	Weekly Frequency	*p* Value	Effect Size	Daily Duration	*p* Value	Effect Size
Gender	MW (*U*)	*U* = 248,292	<0.001	*r* = 0.15	*U* = 236,545	<0.001	*r* = 0.20
Education	K–W (*H*, df = 5)	*H* = 11.76	0.038	η^2^ = 0.01	*H* = 16.07	0.007	η^2^ = 0.01
Employment	K–W (*H*, df = 8)	*H* = 29.16	<0.001	η^2^ = 0.02	*H* = 44.57	<0.001	η^2^ = 0.03
Age	Spearman (ρ)	ρ = 0.04	0.138	—	ρ = 0.06	0.026	—

Note: MW = Mann–Whitney U test; K–W = Kruskal–Wallis test; ρ = Spearman’s rank correlation coefficient. Effect sizes are reported as rank-biserial correlation (*r*) for Mann–Whitney and eta-squared (η^2^) for Kruskal–Wallis. Statistically significant results are highlighted by *p* < 0.05.

**Table 4:** Non-parametric tests for anthropometric characteristics influences on exercise motives.

Variable	Test Type	Statistic	*df*	*p* Value	Effect Size
BMI	K–W (*H*)	*H* = 160.30	4	<0.001	η^2^ = 0.10
Weight history	MW (*U*)	*U* = 185,774.00	—	<0.001	*r* = 0.21

Note: K–W = Kruskal–Wallis test; MW = Mann–Whitney U test; η^2^ = eta-squared; *r* = rank-biserial correlation. Sample sizes varied slightly across analyses due to missing data (*N* = 1541–1542).

**Table 5:** Kruskal–Wallis tests for personality traits across exercise types (*N* = 1543).

Trait	Statistic (*H*, *df* = 3)	*p* Value	Effect Size (η^2^)
Extraversion	21.271	<0.001	0.014
Neuroticism	49.308	<0.001	0.032
Openness	11.908	0.008	0.008
Agreeableness	21.304	<0.001	0.015
Conscientiousness	20.471	<0.001	0.015

Note. K–W = Kruskal–Wallis test; η^2^ = H/(*N* − 1) with *N* = 1543.

**Table 6:** Mean Ranks of Personality Traits Across Exercise Types.

Trait	Cyclists	Runners	Fitness	Swimmers
Extraversion	844.61	803.49	722.74	704.56
Neuroticism	651.31	759.75	804.12	922.17
Openness	776.84	817.89	721.46	789.07
Agreeableness	868.22	769.97	735.78	712.44
Conscientiousness	833.31	814.22	721.06	701.49

Note. Higher mean ranks indicate stronger expression of each trait within the corresponding exercise group.

**Table 7:** Kruskal–Wallis tests for personality traits across exercise motives (*N* = 1541).

Trait	Statistic (*H*, *df* = 4)	*p* Value
Extraversion	2.62	0.623
Neuroticism	1.00	0.910
Openness	4.93	0.295
Agreeableness	5.66	0.226
Conscientiousness	2.55	0.637

Note. None of the traits showed statistically significant variation across motive categories.

**Table 8:** Cluster composition based on demographic, anthropometric, psychological, and behavioral variables.

Cluster	Size (*n*, %)	Age (M, SD)	BMI (M, SD)	Sessions/ Week (M, SD)	Hours/ Day (M, SD)	Extraversion (M, SD)	Neuroticism (M, SD)	Openness (M, SD)	Agreeableness (M, SD)	Conscientiousness (M, SD)
1	695 (45.0%)	38.47 (8.84)	24.85 (2.61)	2.62 (0.77)	2.15 (0.69)	52.54 (7.58)	39.99 (9.87)	49.34 (7.39)	52.98 (6.70)	51.18 (7.15)
2	535 (34.7%)	34.21 (7.95)	21.68 (3.12)	2.38 (0.77)	1.84 (0.64)	51.57 (7.87)	42.18 (9.58)	49.19 (7.47)	52.12 (6.69)	50.28 (7.85)
3	313 (20.3%)	22.17 (2.74)	21.64 (3.09)	2.46 (0.95)	1.90 (0.63)	50.95 (8.00)	42.72 (9.67)	48.56 (7.51)	52.28 (7.12)	50.37 (7.01)

Note. Values for continuous variables are means (M) and standard deviations (SD). Values for categorical variables represent frequencies (*n*). Cluster sizes and profiles are derived from the TwoStep Cluster output (*N* = 1543).

**Table 9:** Distribution of exercise motives across clusters.

Motive Category	Cluster 1	Cluster 2	Cluster 3	Total
Health	85	46	21	152
Enjoyment	522	293	198	1013
Weight loss	50	52	23	125
Appearance	34	140	70	244
Rehabilitation	4	2	1	7
Total	695	535	313	1543

Note. Values represent frequencies (*n*). χ^2^(8) = 127.39, *p* < 0.001, Cramér’s *V* = 0.203.

**Table 10:** Exercise type preferences by cluster.

Exercise Type	Cluster 1	Cluster 2	Cluster 3	Total
Cycling	203	90	37	330
Running	314	118	54	486
Fitness	70	269	198	537
Swimming	108	58	24	190
Total	695	535	313	1543

Note. Values represent frequencies (*n*). χ^2^(6) = 359.13, *p* < 0.001, Cramér’s *V* = 0.341.

## Data Availability

The data presented in this study are available on request from the corresponding author due to privacy, legal, and ethical reasons.

## Abbreviations

The following abbreviations are used in this manuscript:

BMI	Body Mass Index
TPQue5	Traits Personality Questionnaire 5
NEO PI-R	Revised NEO Personality Inventory
BREQ-2	Behavioral Regulation in Exercise Questionnaire-2
VIF	Variance Inflation Factor
